# Bishonokiol A Induces Multiple Cell Death in Human Breast Cancer MCF-7 Cells

**DOI:** 10.31557/APJCP.2020.21.4.1073

**Published:** 2020-04

**Authors:** Hong-Mei Li, Bohan Li, Hui Ma, Xiaolong Sun, Meilin Zhu, Yiqun Dai, Tao Ma, Qiang Huo, Cheng-Zhu Wu

**Affiliations:** 1 *School of Pharmacy, *; 2 *Department of Medicinal Chemistry, School of Pharmacy, Bengbu Medical University, 2600 Donghai Road, Bengbu, Anhui, China. *

**Keywords:** Breast cancer, bishonokiol A, cell death, necroptosis, cyclophilin D

## Abstract

**Objective:**

A dimeric neolignan, bishonokiol A (BHNKA) isolated from *Magnolia grandiflora*, significantly inhibits the proliferation of human breast cancer cells. However, the exact mechanism of BHNKA induced breast cancer cell death is unknown. In this study, we investigated the pharmacological mechanism underlying BHNKA induced MCF-7 cell death.

**Methods:**

Cell viability measurement was performed by the MTT assay. Flow cytometry with PI staining, DAPI staining, and electron microscopy were used to analyze cellular death modes. In addition, western blotting, siRNA transfection, ATP assay, and fluorescence microscopy were used to determine the mechanism of BHNKA induced MCF-7 cell death.

**Results:**

BHNKA induced cell death by apoptosis, necroptosis and autophagy at the same concentration and time in MCF-7 cells, and electron microscopy confirmed these results. The mechanism of BHNKA triggered apoptosis and autophagy in MCF-7 cells was primarily due to an increase in the Bax/Bcl-2 ratio and simultaneous up-regulation of LC3-II protein expression, respectively. BHNKA induced necroptosis by activation of the RIP1-RIP3-MLKL necroptosis cascade, up-regulation of cyclophilin D (CypD) protein expression to stimulate ROS generation. We further demonstrated that siRNA-mediated down-regulation of CypD protected against BHNKA induced cell death.

**Conclusions:**

These results suggest that BHNKA may be a potential lead compound for development as an anti-breast cancer agent for induction of multiple cell death pathways.

## Introduction

Breast cancer is the most common cancer in women, threatening their health and life (Miller et al., 2019). Although chemotherapy shows positive clinical effects in breast cancer, apoptotic drug resistance, substantial side-effects, and dose-limiting toxicity eventually reduce the chemotherapeutic efficacy (Ateba et al., 2018; Holohan et al., 2013; Kotecha et al., 2016). Therefore, research and development effort for developing highly effective and low toxicity anti-breast cancer drugs have become indispensable for saving lives. 

Programmed cell death (PCD), defined as regulated cell death mediated by an intracellular program, plays crucial roles in anti-cancer therapy (Su et al., 2015). Apoptosis and autophagy are considered two types of PCD and serve as important targets for cancer chemotherapy (Levy et al., 2017; Mohamed et al., 2017). Cancer cells are initially sensitive to apoptotic induction, but develop resistance eventually (Pommier et al., 2004). Regulated necrosis, termed as necroptosis, is emerging as a new paradigm of cell death that can be activated when the apoptotic machinery is genetically or pathogenically defective (Degterev et al., 2005). Developing a class of anti-cancer agents that target cancer cells via induction of necroptosis may significantly improve the effectiveness of cancer chemotherapy especially for the drug-resistant (Grassilli et al., 2013; Krysko et al., 2017; Sun et al., 2016; Wu et al., 2013; Xuan et al., 2009).

**Figure 1.**
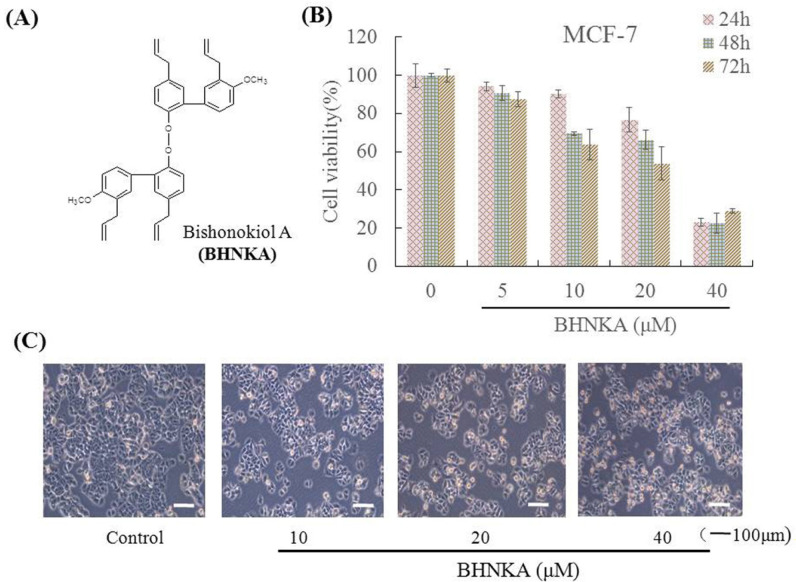
BHNKA Inhibits Proliferation of Human Breast Cancer MCF-7 Cells. (A), Chemical structure of BHNKA. (B), MCF-7 cell treated with different concentration of BHNKA for 24, 48, and 72 h and the cell viability was measured by MTT assay. (C), Cell morphology was examined by light microscopy

Natural products have many advantages, such as structural diversity, unique biological activities, multiple targets, low toxicity, and the ability to circumvent drug-resistance (Wang et al., 2015). Thus, the search for new anti-cancer agents from natural sources has gained importance in recent years. Bishonokiol A (BHNKA) is a dimeric neolignan isolated from *Magnolia grandiflora *seeds, and exhibits anti-proliferative activity, induces apoptosis, and inhibits invasion and migration of cancer cells (Figure 1A) (Li et al., 2015; Li et al., 2019). However, the pharmacological mechanism of BHNKA induced breast cancer cell death is still unclear. In this study, we report the effect of BHNKA on MCF-7 cells and its mechanism underlying breast cancer cell death.

## Materials and Methods

*Reagents and antibodies*

BHNKA was isolated from *M. grandiflora*, and the purity of BHNKA was higher than 96% by HPLC analysis (Li et al., 2015). Dulbecco’s modified Eagle’s medium (DMEM) and 0.25% trypsin were purchased from Hyclone (UT, USA). Fetal bovine serum (FBS) was purchased from Sijiqing (Hangzhou, China). 3-(4,5-Dimethylthiazol-2-yl)-2,5-diphenyl tetrazolium bromide (MTT), dimethyl sulfoxide (DMSO), necrostatin-1 (Nec-1), cyclosporin A (CsA), z-VAD-fmk, chloroquine (CQ), and 4,6-diamidino-2- phenyl indole (DAPI) were purchased from Sigma-Aldrich (St. Louis, MO, USA). Propidium iodide (PI) assay kits, JC-1 kit, ATP kit, and reactive oxygen species (ROS) assay kits were purchased from Beyotime Institute of Biotechnology (Wuhan, China). Lipofectamine 2000 was purchased from Invitrogen (USA). The following antibodies were used: anti-Bax and anti-Bcl-2 antibodies were purchased from Proteintech Group (Chicago, USA); anti-caspase-9 was purchased from Cell Signaling (USA); anti-CypD antibody was purchased from Affinity Biosciences (USA); anti-RIP1, anti-RIP3, anti-MLKL, and anti-LC3 antibodies were purchased from Abcam (Cambridge, MA, UK); anti- GAPDH antibody, goat anti-rabbit and goat anti-mouse secondary antibodies were purchased from BioSharp (Hefei, China).

*Cell line and cell culture*

Human breast cancer MCF-7 cell line was purchased from the Chinese Academy of Sciences Cell Bank (Shanghai, China). The cells were maintained in DMEM containing 15% FBS and 1% penicillin/ streptomycin (Gibco, USA), in an atmosphere containing 5% CO_2_ at 37°C.

*MTT assay*

The cell viability was determined by using MTT assay, as described previously (Li et al. 2019). The MCF-7 cells were seeded in 96-well plate at a density of 6,000 cells per well and treatment with various concentrations of BHNKA for 24, 48, and 72 h. At the end of each time point, MTT solution was added to each well and incubated at temperature without light. After 4 h, the MTT solution was removed, and formazan was dissolved in 100 μl of DMSO for 10 min. The absorbance (A) was determined using a microplate spectrophotometer at 570 nm (Bio-Rad, USA).

*Flow cytometry*

Cells were seeded at a density of 1.2×10^5^ cells/well in 12-well plates. After the cells adhered, they were treated with various concentrations of BHNKA and cultured for 24 h. The cells were collected, washed with PBS, fixed with cold 75% ethanol, and stained with PI and evaluated using Accuri C6 flow cytometry (BD Biosciences, USA).

*DAPI staining*

Cells were seeded in 6-well plates at a density of 2×10^5^ cells/well. Following treatment with various concentrations of BHNKA for 24 h, the cells were washed with PBS and fixed with 4% paraformaldehyde for 15 min at 4°C. Later, the cells were washed with ice-cold PBS and incubated with 500 μl of DAPI solution in the dark for 5 min. After staining, the cells were washed three times with PBS and observed using a fluorescence microscope (Olympus, Tokyo, Japan). 

*Electron microscopy*

Cells in a 10-cm culture dish were incubated for 6 h with vehicle control or BHNKA (40 μM). After incubation, the cells were washed and fixed with 2.5% glutaraldehyde, followed by storage at 4°C. The post-fixed specimens were sent to Servicebio (China) for subsequent processing and observation by transmission electron microscopy (TEM). The processed samples were imaged for observing the submicroscopic cellular structures, as described previously (Wu et al., 2019). 

*Western blotting*

The cells were homogenized in RIPA lysis buffer for 30 min on ice, and cell lysates centrifuged at 12,000 rpm for 30 min at 4°C. The protein concentrations measured using the BCA protein assay kit and the proteins separated on SDS-PAGE and transferred to a PVDF membrane. After blocking with 5% skimmed milk, the membranes were incubated with the primary antibodies (Bax, Bcl-2, RIP1, RIP3, MLKL, CypD, and LC3) overnight at 4^o^C followed by the appropriate secondary antibodies. GAPDH was used as a loading control. An enhanced chemiluminescence kit and gel imaging equipment (Bio-Rad, USA) were used to detect the protein expression. 

*Small interfering RNA (siRNA) transfection*

The CypD siRNAs were obtained from Gene-Pharma (China). Following the manufacturer’s protocol, the siRNAs were transiently transfected into MCF-7 cells in 6-well plates using Lipofectamine 2000 (Invitrogen, USA). The siRNA sequences of used for experiments with human *CypD* were as follows: Negative control sense, 5’-UUCUCCGAACGUGUCACGUTT-3’and antisense, 5’-ACGUGACACGUUCGGAG AATT-3’; Positive control sense, 5’-UGACCUCAACUACAUGGUUTT-3’ and antisense, 5’-AACCAUGUAGUUGAGGUCATT-3’;*CypD-*homo-635 sense, GUGCG UUAUUGCAGA AUGUTT and antisense, ACAUUCUGCAAUAACGCACTT; *CypD-*homo-912 sense, CUGCAACCU AUAGCUUUAATT and antisense, UUA AAGCUAUAGGUUGCAGTT; *CypD-*homo-1139 sense, CCAGGCAGAAUUGCU GAAATT and antisense, UUUCAGCAAUUCUGCCUGGTT.

*Detection of Mitochondrial membrane potential*

MCF-7 cells were seeded in 6-well plates at a density of 2×10^5^ cells/well, and then cultured with various concentrations (10, 20, and 40 μM) of BHNKA for 24 h at 37°C. After 24 h, JC-1 staining solution and culture medium were added in each well, followed by the well mixing and incubation for 20 min. After staining with JC-1, the supernatant was aspirated from the cells, followed by washes with cold JC-1 staining buffer. Following the manufacturer’s protocol, the cells were imaged by fluorescence microscopy. 

*Measurement of ATP levels*

MCF-7 cells were seeded into 12-well plates at a density of 2×10^5^ cells/well, followed by treatment with various concentrations (10, 20, and 40 μM) of BHNKA. After 5 h, the cells were washed, lysed with RIPA lysis buffer on ice, and cell lysates centrifuged at 15,000 rpm for 5 min. The supernatant was harvested for subsequent examination. Following the ATP assay kit’ protocol, ATP test solution and sample were then added to 96-well plates accompanied by well mixing, and ATP levels were determined using a luminoskan luminometer (Thermo Scientific, USA).

*Measurement of ROS production*

The redox-sensitive dye DCFH-DA was used to evaluate the levels of intracellular ROS, as described previously (Wu et al., 2019). MCF-7 cells were seeded in 12-well plates at a density of 2×10^5^ cells/well, followed by treatment with varying concentrations (10, 20, and 40 μM) of BHNKA for 3 h. After incubation, the supernatant was removed, followed by the addition of 1 ml of diluted DCFH-DA (diluted with the serum-free medium to a final concentration of 10 μM) and incubated for 20 min at 37°C. Fluorescence intensity of the oxidized substrate was analyzed by a fluorescence microscope. 

*Statistical analysis*

SPSS v.16.0 software (SPSS Inc., Chicago, IL, USA) was used for data analysis, and t-test was conducted to compare the data between the two test groups. P*<0.05 and P**<0.01 were considered statistically significant.

## Results

*BHNKA exhibits anti-proliferative activity against MCF-7 cells*

First, we analyzed the anti-proliferative activity of BHNKA on human breast cancer MCF-7 cells by treatment with various concentrations (0~40 μM) of BHNKA for 24, 48, and 72 h. BHNKA showed significantly inhibits the proliferation of MCF-7 cells in a concentration- and time-dependent manner, consistent with the previous study (Figure 1) (Li et al., 2019). 

*BHNKA induces apoptosis in MCF-7 cells*

**Figure 2:**
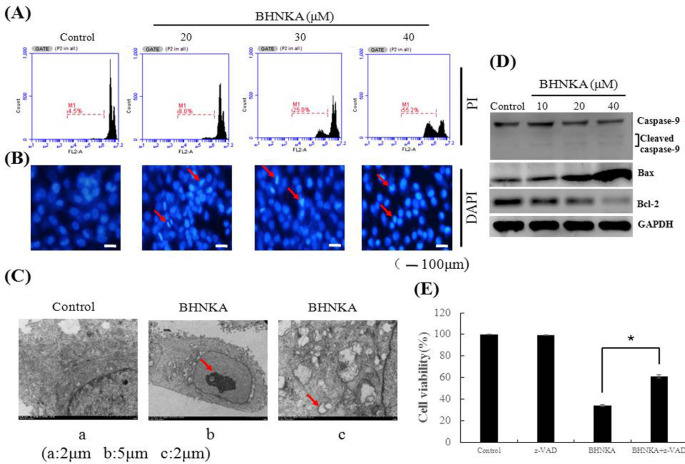
BHNKA Induced Apoptosis in Human Breast Cancer MCF-7 Cells. (A), Cell death was analyzed using flow cytometric with PI staining exposed to various concentration of BHNKA for 24 h. (B), MCF-7 cells were treatment with various concentration of BHNKA for 24 h, subjected to DAPI staining and visualized by fluorescence microscopy. Red arrowheads indicated chromatin concentration and nuclear pyknosis in massive cells. (C), Electron microscopy were used to analyze morphological characteristics of apoptosis. (D), The expression of caspase-9, Bax and Bcl-2 proteins were analyzed by western blotting. GAPDH served as loading control. (E), Cell viability treated with DMSO, z-VAD (20 μM), BHNKA (40 μM), and BHNKA pre-treatment with z-VAD were analyzed by MTT assay. * P < 0.05 compared with the control

The cell death in MCF-7 cells exposed to BHNKA for 24 h was analyzed using flow cytometry with PI staining. As shown in Figure 2A, the cell death rates were 8.0, 25.0, and 55.2% in cells treated with 20, 30, and 40 μM of BHNKA, respectively. DAPI staining of cells exposed to BHNKA showed specific morphological characteristics of apoptosis such as bright nuclear condensation (Figure 2B). To further confirm the mode of cell death induced by BHNKA treatment, we observed the submicroscopic structure of cells under an electron microscope. As shown in Figure 2C, the cells treated with BHNKA exhibited typical characteristics of apoptosis, such as chromatin concentration and nuclear pyknosis in massive cells. Concurrently, with increasing concentrations of BHNKA, slightly greater levels of caleaved caspase-9, proapoptotic protein Bax level gradually increased, and anti-apoptotic protein Bcl-2 decreased (Figure 2D). Furthermore, cell viability after treatment with BHNKA alone was 34.1%, whereas it increased to 60.9% after the combined treatment with BHNKA and the apoptosis inhibitor, z-VAD-fmk (Figure 2E). These results indicated that BHNKA induced apoptosis in MCF-7 cells. 

*BHNKA induces necroptosis in MCF-7 cells*

**Figure 3:**
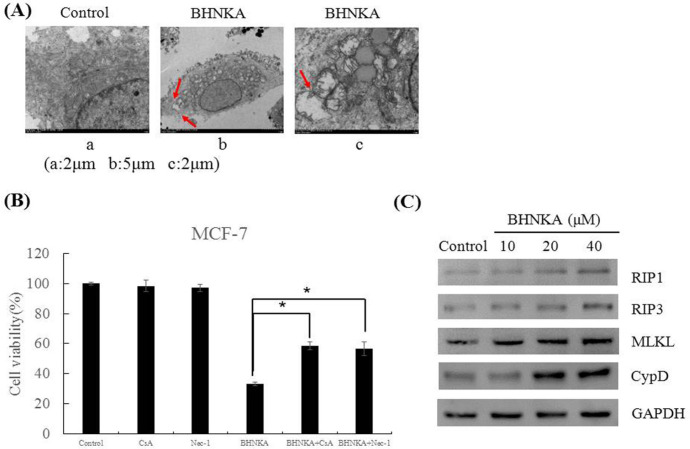
BHNKA Induced Necroptosis in Human Breast Cancer MCF-7 Cells. (A), Morphological characteristics of cells treated with BHNKA (40 μM) for 24h by electron microscope. Red arrowheads indicated cell membrane integrity and swelling of cellular orgenelles. (B), Cell viability following treated with BHNKA (40 μM) with or without pre-treatment with necroptosis inhibitors (20 μM of Nec-1 and CsA) were measured by MTT assay. (C), Western blotting analyses were used to measure the expression of necroptosis-related proteins, such as RIP1, RIP3, MLKL, and CypD. * P < 0.05 compared with the control

Interestingly, we also noticed that BHNKA induced necroptosis at the same concentration and time in MCF-7 cells. The cells treated with BHNKA (40 μM) for 24 h showed morphological characteristics of necrosis as observed by an electron microscopy such as loss of plasma membrane integrity and organelle swelling (Figure 3A). Meanwhile, cell viability after treatment with BHNKA alone was 33.2%, which increased to 56.6% and 58.5% after the combination treatment with BHNKA and the Nec-1 (necroptosis inhibitor) and CsA (CypD inhibitor), respectively (Figure 3B). Furthermore, western blotting showed a significant increase in the necroptosis-related proteins (RIP1, RIP3, MLKL, and CypD) upon BHNKA treatment (Figure 3C). 

*Effect of CypD on necroptosis of MCF-7 cells*

**Figure 4.**
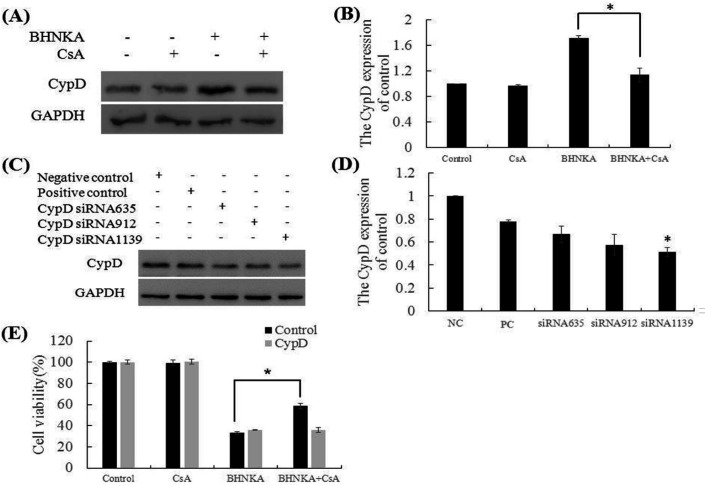
BHNKA Induced Necroptosis in MCF-7 Cells That is Dependent on CypD Expression. (A), CypD inhibitor CsA (20 μM) was added to MCF-7 cells for 24 h prior to the treated with BHNKA, then the expression of CypD was measure by western blotting. (B), Quantification of expression of CypD protein down- regulated by CsA. (C), MCF-7 cells were transfected with CypD siRNA, and whole-cell lysates were subjected to western blotting. (D), Quantification of expression of CypD protein down-regulated by siRNAs. (E), After transfection with CypD siRNA, cells were treatment with BHNKA for 24 h, with or without pre-treatment with CsA. The cell viability was measured by MTT assay. * P < 0.05 compared with the control

Cyclophilin D (CypD) plays a critical role in the regulation of mitochondrial permeability transition and cell death (Baines et al., 2005). The CypD expression was down-regulated significantly in MCF-7 cells after treatment with BHNKA and CsA, compared to treatment with BHNKA alone (Figure 4A and 4B). To explore the role of CypD in cell death, we investigated the effect of CypD knock-down in MCF-7 cells by siRNA. The siRNA1139 significantly silenced *CypD* expression leading to loss of CsA protective ability against BHNKA (Figure 4C-4E). Altogether, these results demonstrated that BHNKA induced necroptosis in MCF-7 cells could be mimicked by down-regulation of CypD. 

*BHNKA induces autophagy in MCF-7 cells*

**Figure 5:**
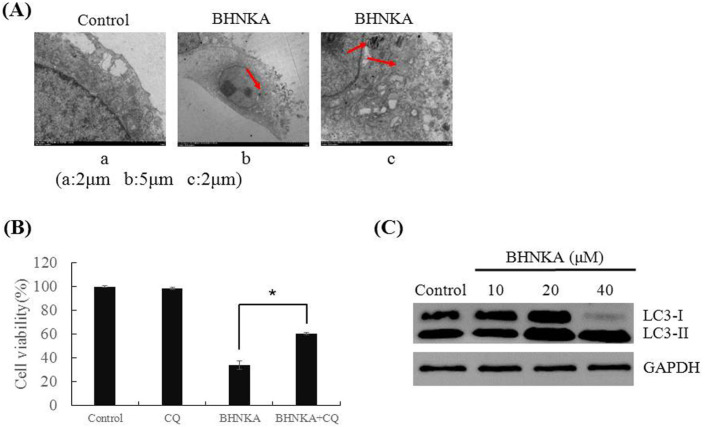
BHNKA Induced Autophagy in Human Breast Cancer MCF-7 Cells. (A), Morphological characteristics of cells treated with BHNKA (40 μM) for 24h by electron microscope. Red arrowheads indicate autophagosome. (B), Cell viability following treated with BHNKA (40 μM) with or without pre-treatment with autophagy inhibitors (20 μM of CQ) were measured by MTT assay. (C), Western blotting analyses was used to measure the expression of autophagy-related proteins, with GAPDH served as loading control. * P < 0.05 compared with the control

BHNKA treated cells exhibited autophagic vacuoles in the cytoplasm, suggesting induction of autophagy in MCF-7 cells, while treatment with the autophagy inhibitor CQ abolished the BHNKA cytotoxicity to MCF-7 cells, as evidenced by the up-regulation of LC3-II protein expression (Figure 5). These data indicated that BHNKA induces autophagy in MCF-7 cells. 

*Effect of BHNKA on mitochondrial function in MCF-7 cells*

**Figure 6:**
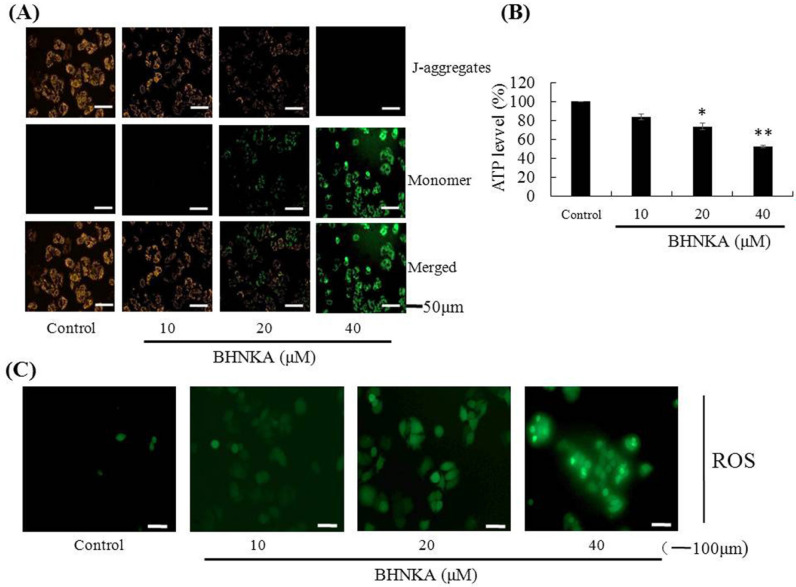
Effect of BHNKA on Mitochondrial Function in MCF-7 Cells. (A), Mitochondrial membrane potential was measured by fluorescence microscopy with JC-1 staining. (B), The cellular ATP levels were determined in MCF-7 cells treatment with various concentrations of BHNKA for 5 h. (C), The ROS levels were measured using the dye DCF after stimulation. * P < 0.05 and ** P < 0.01 compared with the control

To explore whether BHNKA induced cell death is associated with mitochondrial function, we first measured the mitochondrial membrane potential (MMP), as described “Materials and Methods” section. MCF-7 cells were treated with increasing concentrations of BHNKA, and a shift in JC-1 fluorescence from red to green observed (Figure 6A). As shown in Figure 6B, cellular ATP levels decreased to 73.4%% and 52.3%, when treated with 20 and 40 μM of BHNKA, respectively. Measurement of intracellular ROS levels using DCFH-DA showed increased fluorescence intensity of oxidized DCF after treatment with increasing concentrations of BHNKA (Figure 6C). These results indicated that BHNKA triggered MCF-7 cell death is associated with the mitochondrial function. 

## Discussion

Several PCD pathways contribute to cell death, and apoptosis, the classical pathway, has emerged as an important therapeutic target for cancer cells. In cancer chemotherapy, evasion of cell death poses a significant challenge and is frequently associated with failure of cancer cells to undergo apoptosis (Hanahan et al., 2011). Cancer cells are destroyed via various death pathways; however, the mechanism of each pathway differs from one another. Moreover, cancer cells develop pathways to evade apoptosis that are specific to cancer cells only (Hu et al., 2007). For the first time, our results in MCF-7 cells show that BHNKA induced multiple cell death pathways such as apoptosis, necroptosis, and autophagy. 

Necroptosis is a novel type of PCD, which enables significant improvements to target chemotherapy effectiveness against cancer, especially for drug-resistant cancers (Krysko et al., 2017; Sun et al., 2016). Necrostatin-1 is a potent inhibitor of the death domain kinase RIP-1, and specifically inhibits necroptosis and is often used to define necroptosis operationally (Degterev et al., 2005). The various factors that trigger necroptosis in cancer cells include cytokines, RIP1, RIP3, poly(ADP-ribose) polymerase-1 (PARP-1), Ca^2+^, and ROS (Vanlangenakker et al., 2012). Our results showed up-regulated RIP1-RIP3-MLKL signaling pathway and CypD in BHNKA induced process, which exhibits necroptosis characteristics. 

Autophagy is a major intracellular pathway for the degradation and recycling of cytoplasmic contents, damaged organelles, and invasive pathogens, which plays a pivotal role in the pathogenesis of many diseases, such as cancer, neuropathies, and metabolic disorders (Levine et al., 2008). Although it is essential for normal cell function and survival, tumor cells exploit autophagy for treatment resistance. However, autophagy serves as a critical pathway and demonstrates a dual role in the hallmarks of cancer (Singh et al., 2018). Combining standard chemotherapy with an autophagy inhibitor could potentially accelerate cancer cell death, in contrast, activation of autophagy enhances cancer chemotherapy sensitivity and drug-resistance (Mathiasen et al., 2002; Sun et al., 2019). In particular, our results demonstrate that BHNKA induces apoptosis and necroptosis as well as autophagy, and the induced autophagy process is related to an increased expression of LC3-II/I ratio. 

Mitochondria are important organelles for cellular energy and ROS production, and play crucial roles in cell death. As an important effector molecule for apoptosis and necroptosis, CypD is regarded as a key factor for regulating the mitochondrial permeability transition pore function and cell death (Baines et al., 2005; Li et al., 2007; Porter et al., 2018). Consistently, BHNKA-induced necrotic cell death in MCF-7 cells featured a significant up-regulation of *CypD* expression. Down-regulation of CypD by siRNA significantly protected against BHNKA-induced cell death. Our research indicates that BHNKA-triggered PCD is closely associated with changes in mitochondrial function, mainly a decline in the mitochondrial membrane potential, decreased ATP levels, and enhanced ROS generation. 

In summary, the current research showed that BHNKA triggered PCD in MCF-7 cells via induction of apoptosis, autophagy, and necroptosis. In particular, BHNKA induced necroptosis by up-regulating RIP1-RIP3-MLKL signaling pathway and *CypD* expression to stimulate ROS generation. Thus, BHNKA, a potential anti-breast cancer agent, could be used for future clinical applications.

## References

[B1] Ateba SB, Mvondo MA, Ngeu ST, Tchoumtchoua J, Awounfack CF, Njamen D, Kren L (2018). Natural terpenoids against female breast cancer: a 5-year recent research. Curr Med Chem.

[B2] Baines CP, Kaiser RA, Purcell NH (2005). Loss of cyclophilin D reveals a critical role for mitochondrial permeability transition in cell death. Nature.

[B3] Degterev A, Huang Z, Boyce M (2005). Chemical inhibitor of nonapoptotic cell death with therapeutic potential for ischemic brain injury. Nat Chem Biol.

[B4] Grassilli E, Narloch R, Federzoni E 2013) Inhibition of GSK3β bypass drug resistance of p53-null colon carcinomas by enabling necroptosis in response to chemotherapy. Clin Cancer Res.

[B5] Hanahan D, Weinberg RA (2011). Hallmarks of cancer: the next generation. Cell.

[B6] Holohan C, Van Schaeybroeck S, Longley DB (2013). Cancer drug resistance: an evolving paradigm. Nat Rev Cancer.

[B7] Hu X, Han W, Li L (2007). Targeting the weak point of cancer by induction of necroptosis. Autophagy.

[B8] Kotecha R, Takami A, Espinoza JL (2016). Dietary phytochemicals and cancer chemo- prevention: a review of the clinical evidence. Oncotarget.

[B9] Krysko O, Aaes TL, Kagan VE (2017). Necroptotic cell death in anti-cancer therapy. Immunol Rev.

[B10] Li HM, Miao J, Zhu M (2019). Bishonokiol A inhibits breast cancer cell invasion and migration by suppressing hypoxia inducible factor-1α. J Bioenerg Biomembr.

[B11] Li HM, Zhao SR, Huo Q (2015). A new dimeric neolignan from Magnolia grandiflora L. seeds. Arch Pharm Res.

[B12] Li L, Han W, Gu Y (2007). Honokiol induces a necrotic cell death through the mitochondrial permeability transition pore. Cancer Res.

[B13] Levine B, Kroemer G (2008). Autophagy in the pathogenesis of disease. Cell.

[B14] Levy JM, Towers CG, Thorburn A (2017). Targeting autophagy in cancer. Nat Rev Cancer.

[B15] Mathiasen IS, Jäättelä M (2002). Triggering caspase-independent cell death to combat cancer. Trends Mol Med.

[B16] Miller KD, Nogueira L, Mariotto AB (2019). Cancer treatment and survivorship statistics. CA Cancer J Clin.

[B17] Mohamed MS, Bishr MK, Almutairi FM (2017). Inhibitors of apoptosis: clinical implications in cancer. Apoptosis.

[B18] Pommier Y, Sordet O, Antony S (2004). Apoptosis defects and chemotherapy resistance: molecular interaction maps and networks. Oncogene.

[B19] Porter GJ, Beutner G (2018). Cyclophilin D somehow a master regulator of mitochondrial function. Biomolecules.

[B20] Singh SS, Vats S, Chia AY (2018). Dual role of autophagy in hallmarks of cancer. Oncogene.

[B21] Su Z, Yang Z, Xu Y (2015). Apoptosis, autophagy, necroptosis, and cancer metastasis. Mol Cancer.

[B22] Sun W, Bao J, Lin W (2016). 2-Methoxy-6-acetyl-7-methyljuglone (MAM), a natural naphthoquinone, induces NO-dependent apoptosis and necroptosis by H2O2- dependent JNK activation in cancer cells. Free Radic Biol Med.

[B23] Sun X, Yan P, Zou C (2019). Targeting autophagy enhances the anticancer effect of artemisinin and its derivatives. Med Res Rev.

[B24] Vanlangenakker N, Vanden BT, Vandenabeele P (2012). Many stimuli pull the necrotic trigger, an overview. Cell Death Differ.

[B25] Wang S, Fang K, Don G (2015). Scaffold diversity inspired by the natural product evodiamine: discovery of highly potent and multitargeting antitumor agents. J Med Chem.

[B26] Wu C, Gao M, Shen L (2019). Miconazole triggers various forms of cell death in human breast cancer MDA-MB-231 cells. Pharmazie.

[B27] Wu M, Jiang Z, Duan H (2013). Deoxypodophyllotoxin triggers necroptosis in human non-small cell lung cancer NCI-H460 cells. Biomed Pharmacother.

[B28] Xuan Y, Hu X (2009). Naturally-occurring shikonin analogues--a calss of necroptotic inducers that circumvent cancer drug resistance. Cancer Lett.

